# Elevated Levels of Proinflammatory Cytokines in Cerebrospinal Fluid of Multiple Sclerosis Patients

**DOI:** 10.3389/fimmu.2017.00531

**Published:** 2017-05-18

**Authors:** Timur Khaibullin, Vilena Ivanova, Ekaterina Martynova, Georgy Cherepnev, Farit Khabirov, Evgenii Granatov, Albert Rizvanov, Svetlana Khaiboullina

**Affiliations:** ^1^Republican Clinical Neurological Center, Kazan, Russia; ^2^Institute of Fundamental Medicine and Biology, Kazan Federal University, Kazan, Republic of Tatarstan, Russian Federation; ^3^University Kazan Clinic, Kazan Federal University, Kazan, Republic of Tatarstan, Russian Federation; ^4^Nevada Center for Biomedical Research, Reno, NV, USA

**Keywords:** multiple sclerosis, interleukin, cerebrospinal fluid, interferon, C–C motive ligand, C–X–C motive ligand

## Abstract

Multiple sclerosis (MS) is an autoimmune neurodegenerative disease characterized by chronic brain inflammation. Leukocyte infiltration of brain tissue causes inflammation, demyelination, and the subsequent formation of sclerotic plaques, which are a hallmark of MS. Activation of proinflammatory cytokines is essential for regulation of lymphocyte migration across the blood–brain barrier. We demonstrate increased levels of many cytokines, including IL-2RA, CCL5, CCL11, MIF, CXCL1, CXCL10, IFNγ, SCF, and TRAIL, were upregulated in cerebrospinal fluid (CSF), whereas IL-17, CCL2, CCL3, CCL4, and IL-12(p40) were activated in MS serum. Interaction analysis of cytokines in CSF demonstrated a connection between IFNγ and CCL5 as well as MIF. Many cells can contribute to production of these cytokines including CD8 and Th1 lymphocytes and astrocytes. Therefore, we suggest that IFNγ released by Th1 lymphocytes can activate astrocytes, which then produce chemoattractants, including CCL5 and MIF. These chemokines promote an inflammatory milieu and interact with multiple chemokines including CCL27 and CXCL1. Of special note, upregulation of CCL27 was found in CSF of MS cases. This observation is the first to demonstrate CCL27 as a potential contributor of brain pathology in MS. Our data suggest that CCL27 may be involved in activation and migration of autoreactive encephalitogenic immune effectors in the brain. Further, our data support the role of Th1 lymphocytes in the pathogenesis of brain inflammation in MS, with several cytokines playing a central role.

## Introduction

Multiple sclerosis (MS) is a chronic inflammatory disease targeting the central nervous system (CNS). The pathogenesis of MS remains largely unknown. It is believed that nervous tissue damage is due to immune reactivity to myelin basic protein (MBP) (1). The majority of brain lesions are centered near small veins and have prominent lymphocyte and macrophage infiltration (2). The role of inflammation in the development of brain lesions in MS is well documented (3, 4). Evidence suggests that leukocytes cross the blood–brain barrier (BBB) and localize in brain lesions. However, it is still unclear whether inflammation in the brain is initiated within the CNS or is the result of leukocyte migration across the BBB driven by systemic inflammation.

Cerebrospinal fluid (CSF) functions to preserve chemical stability of the brain tissue as well as to remove metabolic toxins and waste. Therefore, the molecular composition of CSF probably closely resembles that of the brain. Analysis of the CSF from MS patients has shown the presence of oligoclonal antibodies, detected as IgG oligoclonal bands, which are commonly used in diagnostic workup for MS. Also, markers of demyelination such as MBP-like material are found in 80% of remitting-relapsing MS (RRMS) patients during acute exacerbations (5). Furthermore, soluble markers of inflammation have been detected in CSF of MS patients; including C9 complement and matrix metalloprotease 9 (6, 7). CSF of MS patients contains high levels of IL-6, TNFα, CCL4, CCL22, CXCL10, and sCD40L suggesting both ongoing inflammation and T cell activation (8, 9). Cytokines are essential for activating immune response and play a pivotal role in establishing and maintaining the inflammatory milieu.

Cytokine profiles of CSF in MS patients are limited and often contradictive (10). For example, studies of IL-6 levels in CSF vary from elevated to being undetectable (8, 11). In addition, Burman and colleagues published data on CSF cytokine profiles that did not agree with the previously demonstrated activation of Th1, Th2, or Th17 responses (9), whereas another study indicated activation of Th1 and Th2 type immune responses (12). In a comprehensive review of PubMed database conducted by Kothur, the leading role of IFN-γ mediated immune response was reported. However, authors note that data on cytokine activation in MS are often inconsistent among different studies (10). Therefore, further investigation is needed to delineate the cytokine profile in CSF and its role in pathogenesis of MS.

Our study of the serum cytokine profile of MS patients revealed upregulation of Th17 cytokines, thus providing conformational evidence of their involvement in MS pathogenesis (13). Also, we observed upregulated levels of serum IL-22 in acute stage RRMS, suggesting that Th22 lymphocytes have a role in MS exacerbation. We extended our investigation to study the cytokine profile of both serum and CSF collected from MS patients. Our data support the concept of CD8+ and Th1 lymphocytes playing a central role in MS brain pathology. Furthermore, in CSF, increased levels of IFNγ were found together with CCL5, MIF, and CCL27. Interaction analysis revealed a strong connection between these cytokines, with IFNγ central to activating CCL5 and MIF. In addition, the CCL5 interaction with CCL27 suggests that these cytokines have a role in brain pathology in MS.

## Materials and Methods

### Study Subjects and Samples

Twenty MS cases, admitted to the Republican Clinical Neurological Center, Republic of Tatarstan, Russian Federation, were diagnosed based upon clinical presentation and brain MRI. Serum and CSF were collected from each patient. In addition, CSF was collected from 20 individuals diagnosed with spinal hernia, and serum was collected from 20 healthy controls. This study was carried out in accordance with the recommendations of Biomedicine Ethic Expert Committee of Republican Clinical Neurological Center, Republic of Tatarstan, Russian Federation with written informed consent from all subjects. All subjects gave written informed consent in accordance with the Declaration of Helsinki. The protocol (No. 218, 11.15.2012) was approved by the Biomedicine Ethic Expert Committee of Republican Clinical Neurological Center, Republic of Tatarstan, Russian Federation.

### Cytokine Analysis

Serum and CSF cytokine profiles were analyzed using Bio-Plex (Bio-Rad, Hercules, CA, USA) multiplex magnetic bead-based antibody detection kits following the manufacturer’s instructions. Bio-Plex Pro Human Cytokine 27-plex Panel and Bio-Plex Human Cytokine 21-plex Panel were used for detection for a total of 48 analytes. Serum and CSF aliquots (50 µl) were used for analysis, with a minimum of 50 beads per analyte acquired. Median fluorescence intensities were measured using a Luminex 200 analyzer. Data collected were analyzed with MasterPlex CT control software and MasterPlex QT analysis software (Hitachi Software, San Bruno, CA, USA). Standard curves for each analyte were generated using standards provided by the manufacturer.

### Detection of Oligoclonal Bands

Oligoclonal IgGs were identified in 10 µl CSF after isoelectric focusing (Hydrasys 2 scan; Sebia, France) and immunofixation with mammalian immunoglobulins directed against human IgG, conjugated to peroxidase (Sebia, France). Then oligoclonal bands were visualized with TTF3 solvent (Sebia, France). Visual analysis and interpretation of presence and number of oligoclonal bands were performed by two experts.

### Statistical Analysis

Descriptive statistics and comparisons of means for normally distributed variables were performed using Statistica 10.0 and XLSTAT software (StatSoft, Inc., Tulsa, OK, USA and Addinsoft, New York, NY, USA, respectively). Non-parametric multiple comparison of medians was conducted using Steel–Dwass All Pairs test that protects the overall error rate (JMP 13.0.0 Software, SAS Institute Inc.).

### Protein Interaction Network Analysis

The Search Tool for the Retrieval of Interacting Genes/Proteins (STRING version 9.0, http://string90.embl.de/) was used to analyze interactions between cytokines with differential expression between MS cases and controls (14). STRING analysis was conducted using high confidence (score 0.7). Cluster analysis was conducted using *k*-means with a value of *k* = 3.

## Results

### Clinical Presentation

Twenty MS cases (5 males and 15 females) were recruited. MS diagnosis was established according to the 2010 Revised MacDonald’s Diagnostic Criteria for MS (15). Sixteen cases were diagnosed with RRMS, one case was diagnosed with primary progressing MS, and three cases were diagnosed with secondary progressing MS (Table 1). The mean age for MS cases was 38 ± 2.8 years (19–55 years), and mean duration of the disease was 48.4 months (2.5–185.6 months). Expanded Disability Status Scale (EDSS) score was 3.2 (1.5–7.5). CSF analysis revealed the presence of oligoclonal bands in 16 MS cases. MRI detected multiple lesions in the subcortical region, corpus callosum, and pons. Thirteen patients received disease-modifying therapy (mainly glatiramer acetate and interferon beta), whereas seven patients received no treatment.

**Table 1:** **Clinical presentation of multiple sclerosis (MS) patients**.

Mean age	38 ± 2.8
Gender (F/M)	15/5
**MS diagnosis**	
Remitting	16
Primary progressing	1
Secondary progressing	3
**Treatment**	
MDT	5
IFNβ	6
Glatiramer acetate	2
None	7

Cerebrospinal fluid cytokine profile was analyzed based on detection of oligoclonal antibody bands and EDDS score, whereas serum cytokine profile was evaluated in MS patients with different duration of the disease. Although some patients received disease-modifying treatment, the therapeutics level in CSF was undetermined. Therefore, the effect of therapeutics on cytokine level in CSF is not analyzed.

### Serum and CSF Cytokine Profile in MS

**Figure 1:**
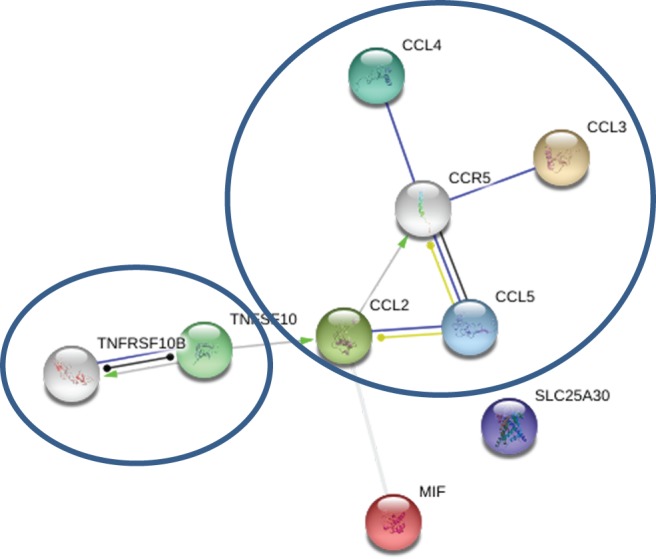
**Serum STRING analysis: green—activation; blue—binding; black—reaction; yellow—expression; white dots are introduced nodes; thicker the line = stronger interaction; STRING 9.0 (http://string90.embl.de/) high confidence 0.7 (circled are clusters *k*-means 3)**.

Out of the 48 cytokines analyzed, levels of only 9 cytokines differed between MS and control serum; IL-17, IL-12(p40), CCL2, CCL3, CCL4, CCL5, CXCL10, MIF, and TRAIL (Table 2). These upregulated cytokines are associated with activation and chemotaxis of leukocytes. Although no clusters could be identified by STRING analysis using only upregulated cytokine data input, when two dots were introduced (CCR5 and TNFRSF10B), two clusters were formed as follows: (1) CCL2, CCL3, CCL4, and CCL5 and (2) TRAIL and TNFRSF10B (Figure 1). Also, a strong association was found between cytokines in each cluster. Analysis of the mode of action suggested that TRAIL activates CCL2, which then upregulates CCR5. CCR5 is positioned in the middle of cluster one and interacts with each member. These data suggest that CCR5 may play a role in MS pathogenesis by supporting an inflammatory milieu.

**Table 2:** **Serum cytokines affected in multiple sclerosis (MS) cases as compared to control**.

Analyte	Control (pg/ml)	MS (pg/ml)
IL-12(p40)	82.3 ± 12.2	530.9 ± 70.8*
CXCL10	720.9 ± 106.7	956.7 ± 136.7
CCL5	377.4 ± 43.3	626.4 ± 81.9*
MIF	153.0 ± 27.0	426.8 ± 126.9
TRAIL	10.7 ± 3.0	98.7 ± 27.6
IL-17	117.3 ± 34.3	371.1.9 ± 38.1
CCL2	498.9 ± 86.3	271 ± 52.1*
CCL3	101.0 ± 16.6	49.0 ± 5.7
CCL4	139.4 ± 24.7	80.7 ± 8.4*

*Serum and cerebrospinal fluid cytokine profiles were analyzed using multiplex magnetic bead-based antibody detection kits. Each serum sample was analyzed in duplicates. Data presented as mean ± SE*.

*Only cytokines that differ in levels between MS cases and healthy control are presented*.

*p < 0.001.

**Figure 2:**
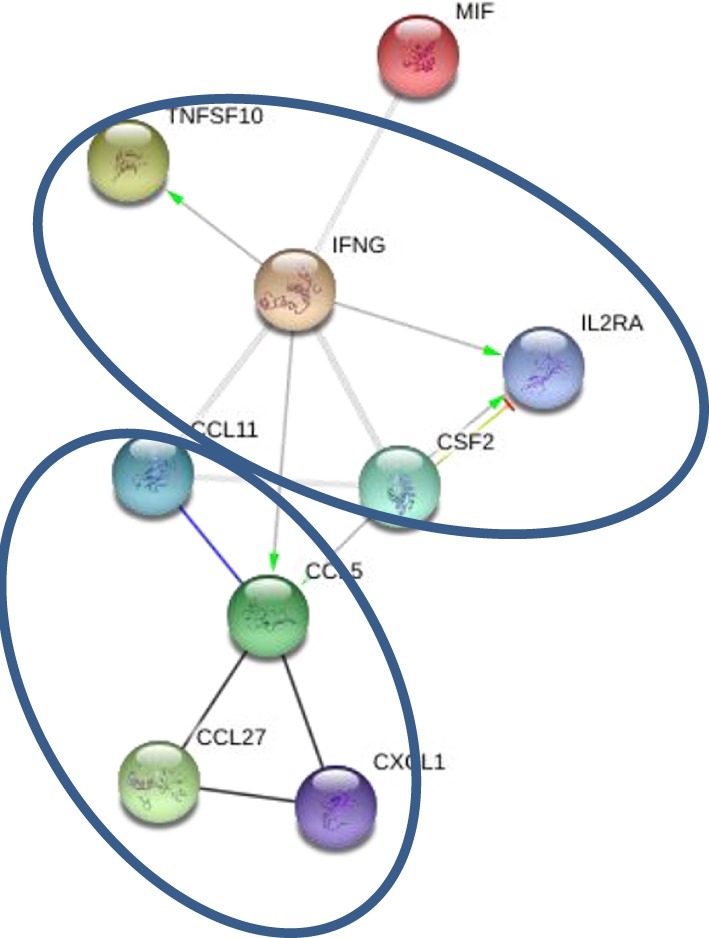
**Interaction between cytokines in cerebrospinal fluid (CSF): green—activation; blue—binding; black—reaction; thicker the line = stronger interaction; STRING 9.0 (http://string90.embl.de/) high confidence 0.7 (circled are clusters *k*-means 3)**.

Levels of 10 cytokines were found increased in CSF of MS cases as compared to controls; IL-2RA, CCL5, CCL11, CXCL1, CXCL10, CXCL12, MIF, IFNγ, TRAIL, and SCF (Table 3). Interestingly, four of these cytokines, CXCL10, CCL5, MIF, and TRAIL were also found to be changed in serum of the MS patients. These cytokines are chemoattractants for T lymphocytes and granulocytes, indicating a role for these leukocytes in MS brain pathology. All cytokines were found to be interconnected when analyzed using the STRING web tool. Two clusters were identified with CCL5, CCL11, CXCL1, CXCL10, CXCL12, and MIF grouped together, whereas IFNγ, TNFSF10, and MIF were separate (Figure 2). A strong association was found between members within each cluster. In addition, there was a strong association between IFNγ and three members of the separate cluster (CCL5, CCL11, and CXCL10), suggesting that there are interactions between these cytokines (Figure 2). Interaction analysis indicates that IFNγ plays a central role, causing activation of TNFSF10, a member of the same cluster, as well as CCL5, which belongs to a separate cluster. Therefore, we suggest that IFNγ is a pivotal participant in the pathogenesis of CNS manifestations of MS.

**Table 3:** **Cerebrospinal fluid (CSF) cytokine affected in multiple sclerosis (MS) cases**.

Analyte	CSF control (pg/ml)	CSF MS (pg/ml)	*P* value
IL-2RA	16.6 ± 2.1	34.5 ± 3.8	0.003
CCL5	16.5 ± 6.1	130.9 ± 27.1	0.005
CCL11	19.9 ± 1.3	47.7 ± 9.1*	0.04
CXCL1	8.8 ± 1.5	21.4 ± 3.1	0.009
CXCL10	509.5 ± 80	1,849.9 ± 478.7*	0.05
CXCL12	21.7 ± 3.5	44.1 ± 6.8*	0.03
IFNγ	102.2 ± 26.2	290.5 ± 65.6*	0.05
MIF	40.7 ± 6.3	272.5 ± 61.8*	0.01
SCF	9.1 ± 0.6	23.5 ± 2.8	0.0009
TRAIL	3.2 ± 0.2	17.3 ± 2.6	0.0004

*Serum and CSF cytokine profiles were analyzed using multiplex magnetic bead-based antibody detection kits. Each serum sample was analyzed in duplicates. Data presented as mean ± SE*.

*Only cytokines that differ in levels between MS cases and healthy control are presented*.

*p < 0.001.

### CSF Cytokine Profile Analysis Based on Presence of Oligoclonal Bands

Detection of IgG oligoclonal bands in CSF of MS cases is commonly used as a part of the diagnostic workup (15). It is believed that oligoclonal IgG bands in CSF are indicative of a B-cell response and inflammation within the CNS (16). Since cytokines play a role in inflammation and regulate immune responses, we sought to determine whether the presence of oligoclonal bands in CSF in MS cases had distinct cytokine characteristics (Table 4). When CSF was analyzed based on the presence or absence of oligoclonal antibody bands, 16 cytokines differed in CSF from MS patients as compared to controls. Values for 13 cytokines were significantly higher in CSF positive for oligoclonal bands, while IL-1b was lower than controls. In CSF samples negative for oligoclonal IgG bands, only four cytokines were found to be upregulated, while levels of G-CSF were lower when compared to controls. Four cytokines were upregulated in oligoclonal band positive and negative samples, CCL5, CCL7, CXCL10, and GM-CSF. Interestingly, two of these cytokines (CCL5 and CXCL10) were found upregulated relative to control when CSF samples were analyzed independent of clinical presentation or presence of oligoclonal bands, suggesting that these cytokines play a fundamental role in disease pathogenesis.

**Table 4:** **Cerebrospinal fluid (CSF) cytokine analysis based on presence of oligoclonal IgG [median (25th–75th percentile)]**.

Cytokine (pg/ml)	Control (*n* = 20)	Oligoclonal antibody positive (*n* = 16)	Oligoclonal antibody negative (*n* = 4)
IL-1b	35.0 (23.1–49.5)	6.6 (4.9–24.2)* 0.02	6.3 (4.7–30.9)
IL-2RA	15.7 (10.3–23.2)	30.3 (23.8–46.7)* 0.003	27.8 (19.4–64.4)
IL-4	2.8 (1.2–6.6)	8.2 (5.7–11.6)* 0.05	7.2 (4.8–9.9)
IL-6	107.7 (75.9–133.0)	45.4 (33.4–97.9)* 0.02	41.1 (26.2–119.3)
IL-7	238.7 (118.8–498.9)	46.2 (36.1–76.1)* 0.001	90.7 (40.9–536.9)
IL-8	1,273.2 (730.7–1,743.2)	219.3 (171.3–547.4)* 0.002	200.1 (116.8–832.2)
IL-12-p40	111.9 (99.8–184.9)	264.4 (213.6–32.4)* 0.001	169.4 (123.9–198.9)
CCL5	6.0 (3.1–29.0)	120.1 (29.6–170.4)* 0.001	92.5 (34.4–216.2)* 0.05
CCL7	0.3 (0.1–0.5)	1.5 (1.2–2.1)* 0.002	2.4 (0.6–15.4)* 0.0003
CCL27	0.2 (0.1–0.3)	27.6 (15.7–45.8)* 0.002	12.6 (0.1–387)
CXCL1	7.7 (6.5–10.3)	20.1 (17.0–23.1)* 0.03	9.9 (4.5–22.2)
CXCL10	562.6 (239.6–717.8)	1,062.3 (429.7–2,008.3)* 0.03	1,147.5 (727.1–2,029.8)* 0.04
G-CSF	86.0 (63.9–135.2)	29.3 (21.9–96.9)	39.1 (23.4–53.8)* 0.03
GM-CSF	54.1 (31.6–186.9)	394.3 (313.3–572.8)* 0.002	389.9 (308.6–462.2)* 0.05
INF-γ	70.6 (52.3–88.8)	192.8 (119.8–298.0)* 0.002	147.3 (130.9–164.1)
MIF	39.4 (24.1–48.3)	140.8 (87.1–291.9)* 0.001	51.7 (22.1–274.8)
SCF	9.6 (6.9–10.6)	26.7 (8.9–37.2)	22.5 (5.9–32.7)* 0.03
TRAIL	3.1 (2.5–3.7)	20.1 (6.5–27.6)* 0.003	5.3 (2.8–27.6)

*P value between control and patient groups.

*Serum and CSF cytokine profiles were analyzed using multiplex magnetic bead-based antibody detection kits. Each serum sample was analyzed in duplicates*.

*Data presented as median (lower quartile–upper quartile). Only cytokines that differ in levels between multiple sclerosis cases and healthy control are presented*.

In should be noted that the level of CCL27 was significantly higher in oligoclonal antibody positive CSF. In addition, the presence of oligoclonal antibodies was accompanied by a significant upregulation of IFNγ, IL-12(p40), and CXCL1, while the levels of these cytokines did not differ between controls and MS cases without oligoclonal bands. Taken together, these data suggest that in MS cases with oligoclonal band positive CSF there is a cytokine profile characteristic for a Th1 immune response [IFNγ and IL-12(p40)] and granulocyte activation (CXCL1 and G-CSF), whereas in CFS negative for oligoclonal IgG there is a lack of the Th1 response, as well as an absence of granulocyte activation. These results suggest that the presence of oligoclonal IgG reflects an ongoing immune process involving diverse leukocyte subsets.

**Figure 3:**
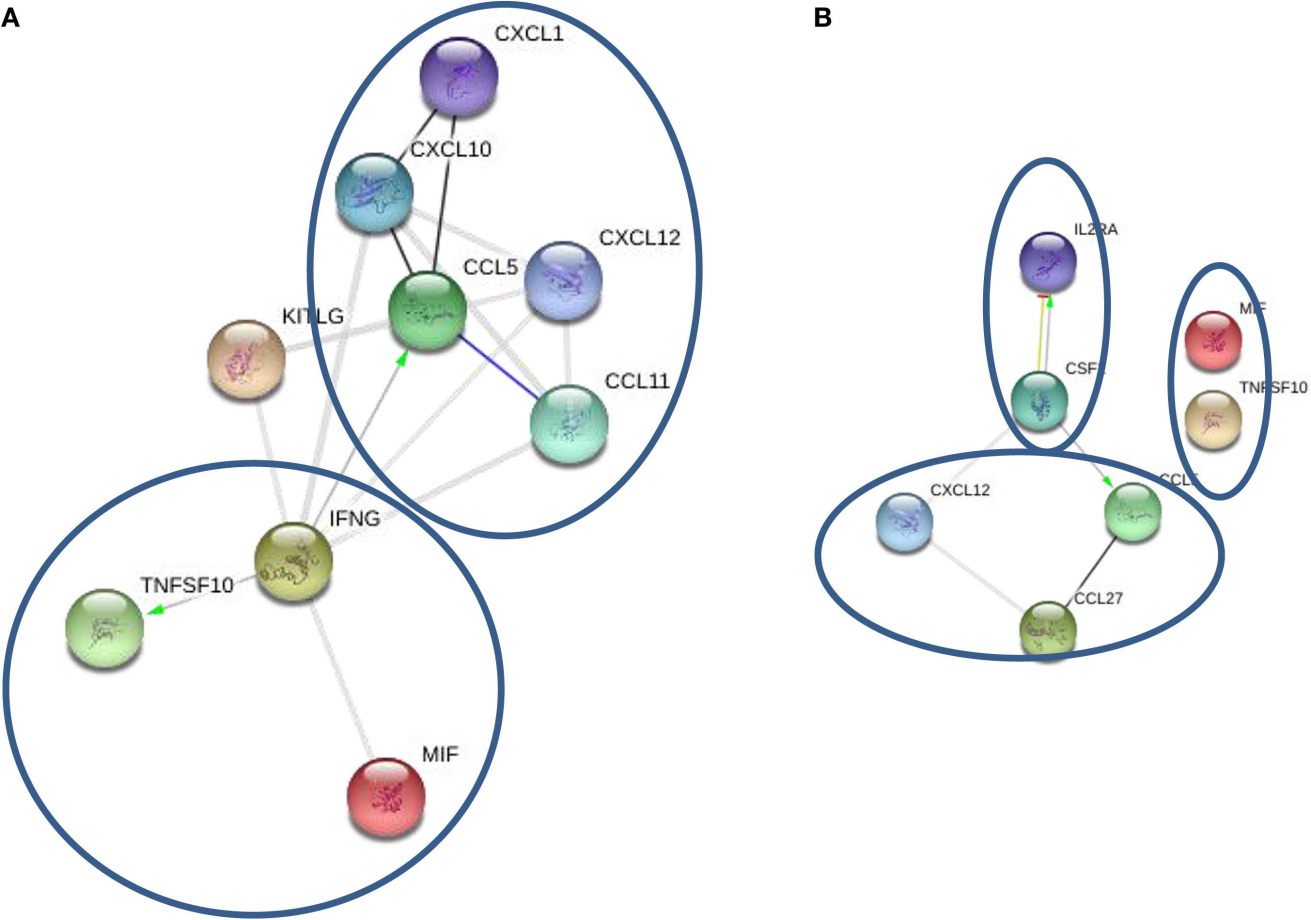
**STRING cytokine analysis of cerebrospinal fluid (CSF) based on the presence of oligoclonal bands**. **(A)** Oligoclonal positive patients (CSF2 = GM-CSF). **(B)** Oligoclonal band negative patients (*P < 0.01; **P < 0.001; ***P < 0.0001).

STRING analysis of cytokine profiles revealed that upregulated cytokines in oligoclonal band positive CSF are interconnected (Figure 3A). Two clusters could be identified with IFNγ, MIF, and TNF10 grouped together, whereas CCL5, CCL11, CCL27, and CXCL1 forming a separate cluster. Interestingly, it appears that activation of IFNγ could cause upregulation of two cytokines within the same cluster as well as CCL5, a member of another cluster. This suggests that IFNγ may play a central role in the pathogenesis of oligoclonal antibody positive MS.

STRING analysis of cytokine profile in patients without oligoclonal bands identified three clusters; however, there were few strong links between members in each cluster (Figure 3B). For example, IL-2RA and GM-CSF were linked forming one cluster, though a link was only detected between CCL5 and CCL27, and there was no link between MIF and TRAIL. Interestingly, it appears that GM-CSF activation was central in the cytokine profile of CSF from oligoclonal band negative patients, as STRING analysis indicated that GM-CSF activation causes upregulation of IL-2RA and CCL5, which interacts with CCL27. Therefore, these data suggest that IFNγ and GM-CSF play an important role in regulation of oligoclonal antibody production in MS CSF.

### CSF Cytokine Profile Analysis Based on EDSS

Expanded Disability Status Scale is used to quantify and monitor changes in the disability in MS over the time and is based on assessment of eight functional systems including pyramidal, cerebellar, brainstem, sensory, bowel/bladder, mental, and visual. EDSS is a 0.5-point step-based scale where patients with 0–1.5 score are characterized as having no disability, whereas scores 2.0 and higher represent various degrees of disability with the highest being death. Since EDSS correlates with the degree of neurological dysfunction, potentially related to the neuroinflammation, we sought to determine whether SCF cytokine profiles vary in patients with different EDSS score. CSF samples were separated into two groups: (1) EDSS ≤ 1.5, representing MS patients without disability and (2) EDSS > 1.5, including MS patients with varying degrees of disability. Twelve cytokines differed in the CSF of MS patients with an average EDSS score of 1.5 when compared to control cases (Table 5). Of these, nine cytokines, namely, IL-1β, IL-2RA, CCL5, CCL7, CCL27, CXCL1, GM-CSF, MIF, and TRAIL, had changed values in patients with oligoclonal bands in their CFS. Importantly, five of these cytokines (IL-2RA, CCL5, CXCL1, MIF, and TRAIL), detailed above, that had changed values in CSF of MS patients independent of clinical presentation or laboratory findings. These data strongly suggest that these cytokines are fundamental to MS pathogenesis.

**Table 5:** **Cerebrospinal fluid (CSF) cytokine analysis based on Expanded Disability Status Scale (EDSS) [median (25th–75th percentile)]**.

Cytokine (pg/ml)	Control (*n* = 20)	Multiple sclerosis (MS) (*n* = 4; pg/ml) (EDSS 1.5 ± 0.03)	MS (*n* = 16; pg/ml) (EDSS 3.7 ± 0.5)
IL-1b	35.0 (23.1–49.5)	5.1 (5.8–6.4)* 0.02	4.7 (6.6–27.8)* 0.04
IL-2	47.1 (37.7–56.1)	20.1 (14.6–36.9)* 0.03	32.5 (21.6–59.4)
IL-6	107.7 (75.9–133.0)	54.9 (27.9–126.2)	45.6 (33.5–106.1)* 0.02
IL-7	238.7 (118.8–498.9)	42.9 (36.8–517.2)	49.0 (36.0–83.5)* 0.02
IL-8	1,273.2 (730.7–1,743.2)	224.5 (174.7–837.1)	231.4 (169.1–549.9)* 0.004
IL-9	60.6 (23.2–82.5)	117.0 (84.8–138.1)* 0.05	89.7 (69.7–100.5)
IL-10	9.0 (8.5–9.9)	13.1 (10.7–33.6)* 0.04	8.8 (7.6–15.1)
IL-12-p40	111.9 (99.8–184.9)	272.9 (169.4–325.1)	243.7 (204.7–310.0)* 0.002
CCL5	6.0 (3.1–29.0)	138.9 (75.4–171.8)* 0.02	98.1 (26.5–168.7)* 0.003
CCL7	0.3 (0.1–0.5)	2.5 (2.1–15.4)* 0.03	1.4 (1.1–1.8)* 0.0003
CCL27	0.2 (0.1–0.3)	35.8 (32.3–387.0)* 0.03	26.5 (14.0–49.3)* 0.003
CXCL1	7.7 (6.5–10.3)	22.2 (18.9–25.9)* 0.05	20.1 (13.4–21.9)* 0.009
CXCL10	562.6 (239.6–717.8)	1,015.5 (711.8–1,310.5)	1,123.1 (321.8–2,252.7)* 0.04
G-CSF	86.0 (63.9–135.2)	69.6 (35.8–238.9)	25.4 (21.4–62.3)* 0.02
GM-CSF	54.1 (31.6–186.9)	425.8 (344.6–481.3)* 0.04	373.1 (301.1–601.6)* 0.002
INF-γ	70.6 (52.3–88.8)	221.5 (152.9–701.1)	128.7 (119.6–298.0)* 0.005
MIF	39.4 (24.1–48.3)	376.8 (271.8–802.3)* 0.03	128.9 (66.4–192.5)* 0.001
SCF	9.6 (6.9–10.6)	28.6 (22.5–37.0)	26.2 (8.6–35.1)* 0.03
TRAIL	3.1 (2.5–3.7)	16.5 (5.3–27.6)* 0.03	(6.5–27.6)* 0.003

*P value between control and patient groups.

*Serum and CSF cytokine profiles were analyzed using multiplex magnetic bead-based antibody detection kits. Each serum sample was analyzed in duplicates*.

*Data presented as median (lower quartile–upper quartile). Only cytokines that differ in levels between MS cases and healthy control are presented*.

Levels of 16 cytokines [IL-1b, IL-2RA, IL-6, IL-7, IL-12(p40), CCL5, CCL7, CCL27, CXCL1, CXCL10, IFNγ, G-CSF, GM-CSF, MIF, TRAIL, and SCF] were changed in patients with EDSS 3.7. Interestingly, values for eight of these cytokines, such as IL-2RA, CCL5, CXCL1, CXCL10, IFNγ, MIF, TRAIL, and SCF, were also altered in MS CSF, for samples analyzed independent of the patient’s disability status. Taken together, these results further suggest that a group of cytokines including IL-2RA, CCL5, CCL11, CXCL1, CXCL10, CXCL12, IFNγ, MIF, CSF, and TRAIL play a role in pathogenesis of MS.

### Serum and CSF Cytokine Analysis Based on the Disease Duration

Serum and CSF samples were grouped according to disease duration and designated as group 1: soon after the diagnosis (4.2 ± 0.8 months after the diagnosis) or group 2: later after diagnosis (76.6 ± 14.3 months after the diagnosis). Six cytokines were upregulated in serum of group 1 MS, while levels of 11 cytokines differed in group 2 MS (Table 6) as compared to control. Interestingly, a larger number of cytokines were affected in CSF of group 1 MS as compared to that in serum of the same patients (15 vs 5). In group 2 MS, CSF level of 9 cytokines remained altered, whereas more cytokines, total of 11, were affected in serum. CSF cytokine profile in group 1 MS cases may indicate activation and chemotaxis of lymphocytes, which continues later in the disease. Of note, upregulation of IFNγ was detected in group 1 CSF samples, while level of this cytokine in serum remained similar to controls. Furthermore, STRING analysis data suggested that IFNγ plays a central role in pathogenesis of both oligoclonal IgG in CSF and EDSS.

**Table 6:** **Cytokine profile of multiple sclerosis serum and cerebrospinal fluid (CSF) collected at different time points after the diagnosis**.

Analyte	Serum (pg/ml)			CSF (pg/ml)		
	Control (*n* = 20)	Group 1 (*n* = 8)	Group 2 (*n* = 12)	Control (*n* = 12)	Group 1 (*n* = 8)	Group 2 (*n* = 12)
IL-1a	2.9 ± 0.3	3.5 ± 0.6	10.1 ± 0.5**	1.5 ± 0.2	3.7 ± 2.1	2.4 ± 0.5
IL-1b	7.6 ± 1.4	5.6 ± 2.5	87.4 ± 15.0	38.0 ± 5.3	11.6 ± 4.3**	7.7 ± 2.0**
IL-2RA	22.8 ± 3.2	25.1 ± 6.2	63.9 ± 4.4**	16.6 ± 1.9	37.9 ± 6.3**	59.2 ± 19.1*
IL-1RA	19.5 ± 4.4	13.1 ± 8.4	8.9 ± 8.4	11.2 ± 1.0	100.8 ± 16.8	190.5 ± 68.6
IL-2	138.8 ± 18.5	124.3 ± 36.1	134.6 ± 34.2	49.2 ± 5.9	26.7 ± 2.8**	98.1 ± 56.3*
IL-3	34.8 ± 8.2	24.1 ± 11.2	62.6 ± 6.9	33.4 ± 3.5	20.4 ± 1.5**	27.7 ± 3.7
IL-4	23.2 ± 4.4	22.1 ± 5.8	76.9 ± 7.9*	3.8 ± 0.8	10.0 ± 2.0**	21.9 ± 7.2*
IL-5	156.7 ± 36.9	124.4 ± 74.9	116.8 ± 12.3	74.8 ± 10.0	27.2 ± 3.1**	248.3 ± 185.6
IL-6	103.4 ± 9.9	78.9 ± 19.5	76.0 ± 17.2	121.2 ± 23.3	35.1 ± 3.1**	159.7 ± 98.0
IL-7	12.6 ± 2.3	7.2 ± 5.3	210.2 ± 13.0	343.7 ± 93.2	46.5 ± 13.7*	199.4 ± 144.4
IL-8	60.3 ± 9.8	25.1 ± 13.4	18.1 ± 15.2	124.1 ± 20.5	15.8 ± 2.2**	136.0 ± 57.7
IL-9	23.6 ± 2.5	26.4 ± 2.4	29.6 ± 2.0	58.0 ± 11.2	84.7 ± 11.1	32.8 ± 21.0
IL-10	163.9 ± 37.5	202.2 ± 69.5	89.6 ± 31.0	9.3 ± 0.9	9.8 ± 1.4	30.5 ± 11.2
IL-12(p40)	82.3 ± 6.2	144.3 ± 12.2**	1,009.7 ± 301.1*	131.4 ± 17.7	214.1 ± 24.5*	229.9 ± 30.1*
IL-12(p70)	361.9 ± 61.5	302.6 ± 110.6	196.4 ± 31.0	33.6 ± 3.9	34.1 ± 4.6	270.1 ± 184.5
IL-13	12.1 ± 2.3	15.7 ± 2.6	11.3 ± 30.3	3.6 ± 4.5	2.6 ± 0.9	4.7 ± 0.2
IL-15	26.1 ± 12.9	28.9 ± 8.7	21.6 ± 9.3	36.4 ± 5.9	28.1 ± 3.7	75.7 ± 48.3
IL-16	179.4 ± 37.9	108.3 ± 74.2	123.9 ± 45.3	64.6 ± 6.9	53.6 ± 9.5	56.6 ± 10.9
IL-17	371.1 ± 54.6	322.4 ± 50.4	266.1 ± 57.7	119.5 ± 31.5	154.3 ± 33.5	320.5 ± 178.5
IL-18	7.9 ± 2.3	21.7 ± 14.2	29.8 ± 0.8**	5.2 ± 0.7	64.2 ± 57.5	56.7 ± 10.9
CCL2	498.9 ± 83.0	457.6 ± 111.3	360.0 ± 141.6	521.2 ± 66.6	391.8 ± 71.7	493.9 ± 59.6
CCL3	101.0 ± 15.9	62.7 ± 23.8	48.9 ± 9.6	11.9 ± 2.4	13.5 ± 2.8	36.6 ± 14.7
CCL4	139.4 ± 23.8	92.8 ± 13.7	80.8 ± 14.5	75.7 ± 11.2	41.5 ± 5.3*	73.6 ± 22.2
CCL5	377.4 ± 41.6	787.5 ± 50.5**	696.5 ± 26.9*	16.5 ± 6.4	119.7 ± 27.5*	162.0 ± 41.9**
CCL7	1,210 ± 160	2,080 ± 180	44.3 ± 1.8*	0.3 ± 0.1	13.1 ± 9.8	19.9 ± 8.5*
CCL11	777.4 ± 165.0	838.7 ± 262.4	556.3 ± 364.9	19.9 ± 2.1	45.0 ± 12.7	72.0 ± 14.3
CCL27	168.2 ± 11.1	171.9 ± 17.0	445.6 ± 18.3**	0.2 ± 0.1	92.2 ± 44.8*	65.8 ± 18.4**
CXCL1	44.4 ± 8.2	94.8 ± 9.5	41.1 ± 11.8	8.8 ± 1.7	98.4 ± 80.0	204.3 ± 128.9
CXCL9	124.7 ± 14.8	85.0 ± 25.3	103.4 ± 36.4	35.5 ± 4.2	76.3 ± 24.3	137.0 ± 70.3
CXCL10	720.9 ± 102.6	705.2 ± 96.9	577.6 ± 72.5	509.5 ± 69.3	2,113.8 ± 1,082.2	1,795.4 ± 606.2
CXCL12	58.6 ± 9.3	48.3 ± 10.0	36.4 ± 14.9	21.7 ± 3.5	46.5 ± 11.9	63.8 ± 11.9
FGF	109.3 ± 19.8	103.0 ± 23.6	68.9 ± 36.3	113.8 ± 25.0	114.3 ± 20.0	129.7 ± 35.2
G-CSF	43.9 ± 7.7	30.9 ± 11.1	34.7 ± 9.2	96.4 ± 14.5	64.6 ± 17.6	419.2 ± 254.3
GM-CSF	117.9 ± 22.4	103.6 ± 25.9	149.5 ± 17.6	134.6 ± 24.0	356.8 ± 27.6	990.6 ± 517.9
HGF	57.0 ± 12.9	78.2 ± 6.1	133.7 ± 6.0	205.0 ± 16.1	172.1 ± 41.7	243.9 ± 45.2
IFNα	26.5 ± 1.4	23.6 ± 2.8	16.6 ± 2.9	44.9 ± 6.6	30.7 ± 1.2	68.3 ± 43.8
IFNγ	134.2 ± 33.9	88.5 ± 37.2	108.2 ± 67.9	102.2 ± 28.6	236.6 ± 33.0**	458.1 ± 122.5*
LIF	26.0 ± 3.0	48.4 ± 23.0	51.6 ± 0.4*	2.5 ± 0.3	2.9 ± 0.7	14.3 ± 11.3
M-CSF	28.1 ± 5.1	107.0 ± 8.1***	196.1 ± 20.1***	71.5 ± 8.0	51.4 ± 12.2	115.2 ± 77.3
MIF	175.0 ± 31.2	268.5 ± 41.5	505.8 ± 63.1	40.7 ± 7.6	248.6 ± 63.1**	256.4 ± 93.5*
NGF-b	1.8 ± 0.2	1.6 ± 0.2	1.5 ± 0.1	1.2 ± 0.2	1.9 ± 0.3	1.5 ± 0.2
PDGF-bb	1,046.3 ± 131.4	1,060.7 ± 198.3	1,008.7 ± 187	14.2 ± 4.4	25.3 ± 13.7	54.7 ± 17.9
SCF	40.6 ± 3.5	47.6 ± 5.9	34.3 ± 7.4	9.0 ± 0.9	19.1 ± 5.2	28.9 ± 2.8
SCGF-b	715.3 ± 125.1	625.5 ± 121.4	1,044.6 ± 88.0	5,699.9 ± 804.5	5,606.2 ± 1,355.4	3,718.8 ± 715.5
TNFβ	2.1 ± 0.2	3.5 ± 0.3*	3.4 ± 0.2*	0.5 ± 0.1	2.3 ± 0.7**	12.2 ± 6.2**
TRAIL	10.7 ± 1.9	25.7 ± 4.2*	28.0 ± 6.6	3.2 ± 0.3	15.7 ± 4.1**	19.8 ± 3.4**
TNFα	27.3 ± 5.5	27.0 ± 10.8	24.7 ± 2.3	5.3 ± 1.4	2.1 ± 1.7	9.1 ± 3.9
VEGF	36.2 ± 4.8	37.2 ± 7.9	29.2 ± 6.0	120.1 ± 30.4	102.5 ± 7.4	450.2 ± 242.3

*P < 0.01; **P < 0.001; ***P < 0.0001 between patient and control groups.

*Group 1—samples collected 4.2 ± 0.8 months after the diagnosis*.

*Group 2—samples collected 76.6 ± 14.3 months after the diagnosis*.

*Serum and CSF cytokine profiles were analyzed using multiplex magnetic bead-based antibody detection kits. Each serum sample was analyzed in duplicates. Data presented as mean ± SE*.

## Discussion

Multiple sclerosis is an autoimmune disease characterized by demyelination and lymphocyte infiltration of brain tissue. It has been suggested that myelin specific T lymphocytes play a role in disease pathogenesis. Studies have shown that these T cells have a Th1 helper inflammatory lymphocyte phenotype, as they produce IFNγ and TNFα when exposed to MBP (17–19). In addition to Th1 lymphocytes, a subset of TH17 lymphocytes has been implicated in the pathogenesis of MS, since the occurrence of Th17 cells was significantly higher during an exacerbation (20, 21). Activation of Th1 and Th17 lymphocytes is orchestrated by a distinct set of cytokines, where IFNγ and IL-12 are essential for developing Th1, while IL-17 is produced by Th17 helper cells (22). Our data support the hypothesis of Th1 and Th17 activation in MS. However, we have demonstrated that CSF and serum cytokine profiles differ, with increased IFNγ and IL-12 in CSF suggesting upregulation of Th1 cells in the brain, while a high serum level of IL-17 indicates Th17 activation in the circulation. STRING analysis further supports the central role of Th1 in brain pathology, as IFNγ, the main Th1 cytokine, was shown to activate several cytokines. The role of Th1 immune response in the MS brain pathology is also corroborated by the results of STRING analysis of CSF cytokine profiles based on presence of oligoclonal IgG. Interaction studies identified IFNγ as the cytokine activating several other molecules including CCL5. Therefore, we suggest that Th1 type response plays an important role in MS brain pathogenesis.

IFNγ has previously been suggested to play an important role in pathogenesis of MS. For example, Lees et al. have shown that interaction between IFNγ and the CNS can modulate neuroinflammation and leukocyte infiltration (23). These authors demonstrated that IFNγ produced by effector T cells was selectively anti-inflammatory in the cerebellum and brainstem. However, IFNγ–IFNγR interactions were required for inflammatory infiltration of the spinal cord. These data demonstrate discrete regional responses to IFNγ during neuroinflammation. In another study, the role of IFNγ in MS pathogenesis was demonstrated by exogenous administration of IFNγ, which increased the exacerbations of MS (24). In addition, Maxeiner et al. have shown that upregulation of IFNγ in CSF of MS cases (25). Our data support the notion that IFNγ is an essential component of MS brain pathology, as levels of IFNγ were higher in CSF of MS cases compared to controls. Furthermore, STRING analysis demonstrated interactions between IFNγ and CCL5 as well as MIF. This suggests that IFNγ effects are associated with activation of CCL5 and MIF.

CCL5 and MIF are secreted by different cell populations within the brain. For example, IFNγ activates CCL5 production by glial cells. CCL5 protects astrocytes and promotes glial cell proliferation and survival (26, 27). Glial cell function is not limited to providing a nurturing environment for neurons; they also play an important role in CNS immunity. For instance, astrocytes can attract T cells within the CNS *via* secretion of multiple chemokines including IL-8, CCL2, CCL5, and CXCL10 (28). Also, upon IFNγ stimulation, astrocytes support proliferation of myelin oligodendrocyte glycoprotein-specific CD4+ T cells (29, 30). In addition, acting like professional antigen-presenting cells, astrocytes can activate encephalitogenic CD4+ T cells through the classical MHC class II pathway (31). Therefore, CNS astrocytes are a plausible source of CCL5, being secreted by glial cells upon IFNγ stimulation. Although the role of CCL5 in brain pathology in MS remains largely unknown, evidence suggests that CCL5 may contribute to the severity of MS (9, 32–34).

MIF is secreted (35) by IFNγ stimulated leukocytes, including lymphocytes, macrophages, dendritic cells, and neutrophils (36, 37). In addition, within the CNS, astrocytes can produce MIF as it has been shown by Choi et al. (38). Interestingly, increased CSF level of MIF in MS cases was reported by Niino et al. (39). Later, Cox et al. confirmed the role of MIF in MS pathogenesis by demonstrating that MIF-deficient mice present with reduced experimental autoimmune encephalomyelitis (EAE) severity and exhibit a lower degree of the CNS inflammation (40). In addition, intraspinal injection of MIF resulted in upregulation of inflammatory mediators in microglia and was sufficient to restore EAE-mediated inflammatory pathology in MIF-deficient mice. Genetic polymorphism studies also support the role of MIF in MS pathogenesis. It has been shown that MIF-173 GC genotype was association with a higher EDSS in MS (41). In another study, patients with MIF-173 CC genotype were shown to have a significantly lower age of onset compared with those with the MIF-173 CG and MIF-173 GG genotypes (42). Our data support the role of MIF in pathogenesis of MS. We believe that IFNγ causes secretion of MIF by leukocytes within the brain of MS patients.

We have demonstrated upregulation of CCL27 in both serum and CSF of MS. These data corroborate our previous publication, where significant upregulation of CCL27 was found in serum of RRMS cases when compared to controls (13). CCL27 has been implicated in an inflammatory allergic reaction, primarily due to homing memory T cells in the skin (43). However, recently, a role for CCL27 in allergic reactions within the CNS has been suggested. Gunsolly et al. have demonstrated expression of CCL27 in the cerebral cortex and limbic regions of the CNS in mice exposed to ovalbumin (44). During the ovalbumin-caused allergic inflammation, CCL27 upregulation was accompanied by infiltration of T cells (44). It is possible that CCL27 targets astrocytes and neurons of the hippocampus, since the expression of CCR10, the CCL27 receptor, was found on these cells (45, 46). CCL27 acts as chemoattractant for antigen-specific T lymphocytes (47); therefore, CCL27 may act to facilitate autoreactive T lymphocyte migration into brain tissue of MS patients promoting brain inflammation.

Together, our data suggest a central role for IFNγ in brain inflammation in MS. We propose that IFNγ could act on astrocytes by releasing a novel subset of chemokines facilitating an inflammatory milieu and promoting migration of autoreactive encephalitic T lymphocytes. Among these cytokines, CCL5, MIF, and CCL27 are of particular interest, since CCL5 and MIF could be activated by IFNγ and known to be secreted by astrocytes. CCL27 is a novel chemokine, whose role in MS pathogenesis is yet to be established.

## Ethics Statement

This study was carried out in accordance with the recommendations of Biomedicine Ethic Expert Committee of Republican Clinical Neurological Center, Republic of Tatarstan, Russian Federation with written informed consent from all subjects. All subjects gave written informed consent in accordance with the Declaration of Helsinki. The protocol (No. 218, 11.15.2012) was approved by the Biomedicine Ethic Expert Committee of Republican Clinical Neurological Center, Republic of Tatarstan, Russian Federation.

## Author Contributions

TK: recruiting patients and collecting informed consent. VI: multiplex analysis and statistical analysis. EM: multiplex analysis and writing the initial draft of the manuscript. GC: statistical analysis. FK: collecting patient specimen; storage and labeling according to the ethic committee standards. EG: primary care physician stratifying patients and controls. AR: intellectual contribution to developing experimental design and trouble shooting. SK: instigating the main scope of the review and intellectual contribution in discussion of the review progress with team of authors.

## Conflict of Interest Statement

The authors declare that the research was conducted in the absence of any commercial or financial relationships that could be construed as a potential conflict of interest.
